# Spectroscopic Study of Methylene Blue Interaction with Coenzymes and its Effect on Tumor Metabolism

**DOI:** 10.17691/stm2025.17.1.02

**Published:** 2025-02-28

**Authors:** D.V. Pominova, A.V. Ryabova, A.S. Skobeltsin, I.V. Markova, I.D. Romanishkin

**Affiliations:** PhD, Senior Researcher, Laser Biospectroscopy Laboratory, Light-Induced Surface Phenomena Department, Natural Sciences Center; Prokhorov General Physics Institute of the Russian Academy of Sciences, 38 Vavilov St., Moscow, 119991, Russia; Associate Professor, Department 87 “Laser Micro-, Nano-, and Biotechnologies, Engineering Physics Institute for Biomedicine”; National Research Nuclear University MEPhI, 31 Kashirskoye Highway, Moscow, 115409, Russia; Senior Researcher, Laser Biospectroscopy Laboratory, Light-Induced Surface Phenomena Department, Natural Sciences Center; Prokhorov General Physics Institute of the Russian Academy of Sciences, 38 Vavilov St., Moscow, 119991, Russia; Associate Professor, Department 87 “Laser Micro-, Nano-, and Biotechnologies, Engineering Physics Institute for Biomedicine”; National Research Nuclear University MEPhI, 31 Kashirskoye Highway, Moscow, 115409, Russia; Junior Researcher, Laser Biospectroscopy Laboratory, Light-Induced Surface Phenomena Department, Natural Sciences Center; Prokhorov General Physics Institute of the Russian Academy of Sciences, 38 Vavilov St., Moscow, 119991, Russia; Senior Teacher, Department 87 “Laser Micro-, Nano-, and Biotechnologies, Engineering Physics Institute for Biomedicine”; National Research Nuclear University MEPhI, 31 Kashirskoye Highway, Moscow, 115409, Russia; PhD Student; National Research Nuclear University MEPhI, 31 Kashirskoye Highway, Moscow, 115409, Russia; Engineer, of Laser Biospectroscopy Laboratory, Light-Induced Surface Phenomena Department, Natural Sciences Center; Prokhorov General Physics Institute of the Russian Academy of Sciences, 38 Vavilov St., Moscow, 119991, Russia; Junior Researcher, Laser Biospectroscopy Laboratory, Light-Induced Surface Phenomena Department, Natural Sciences Center; Prokhorov General Physics Institute of the Russian Academy of Sciences, 38 Vavilov St., Moscow, 119991, Russia

**Keywords:** tumor metabolism, time-resolved fluorescence microscopy, methylene blue, spectroscopy, NADH and FADH_2_ coenzymes, lactate

## Abstract

**The aim of the investigation** is to study the interaction of methylene blue (MB) with NADH, FADH_2_ coenzymes and lactate, and to evaluate a long-term effect of its intravenous or oral introduction on tumor metabolism *in vivo*.

**Materials and Methods:**

The MB interaction with NADH, FADH_2_ coenzymes and lactate was studied using absorption spectrophotometry. A long-term effect of MB on tumor metabolism *in vivo* was investigated on a mice model of Ehrlich carcinoma. The effect of MB on tumor metabolism *in vivo* was assessed using time-resolved fluorescence microscopy based on the NADH fluorescence lifetime.

**Results:**

NADH has been established to be the main coenzyme with which MB interacts. The reduction of the lactate quantity is mediated by the shift of tumor metabolism as a result of MB interaction with the NADH. In the experiments *in vivo*, no noticeable tumor growth rate reduction was observed in the groups with intravenous MB introduction in comparison with the control. In the group receiving MB with drinking water, a decrease of the tumor growth rate, reduction of oxygenation level, and a1/a2 metabolic index were observed, which confirms the shift from glycolysis to oxidative phosphorylation.

**Conclusion:**

The possibility of using MB for the tumor metabolism correction and growth rate reduction has been demonstrated, however, the time of therapy and MB concentration should be optimized to obtain more pronounced therapeutic effect.

## Introduction

Methylene blue (MB) is a thiazine dye synthesized first in 1876. Initially, MB was used in microscopy as a staining agent [1]. Owing to its redox properties, it has been used for a long time as cyanide, carbon monoxide, and hydrogen sulfide poisoning antidote [2–4], and in treatment of methemoglobinemia [5, 6]. With the emergence of other antidotes, interest in MB has waned for a long time.

In the last years, there is growing interest in using the redox properties of MB for treating neurodegenerative diseases [7] such as Alzheimer’s, Parkinson’s, Huntington’s diseases, and amyotrophic lateral sclerosis characterized by metabolic dysfunction. The ability of MB to generate reactive oxygen species together with a small molecular size makes this dye attractive for using it as a photosensitizer in photodynamic therapy [8, 9].

The mechanism of MB action as an antidote and in neurodegenerative diseases is similar: in low doses, MB stimulates mitochondrial breathing delivering electrons to the electron transport chain. In this case, MB can receive an electron from NADH in the presence of complex I and donate it to cytochrome *c* providing an alternative pathway of electron transport bypassing complexes II and III. As a result, MB can increase oxygen consumption, decrease glycolysis and increase glucose uptake *in vitro.* In addition, MB may be an acceptor of electrons coming out from complexes II and III, thereby minimizing the formation of superoxide anion and other reactive oxygen species and reducing the oxidative stress [10].

Reprogramming of energy metabolism is a common feature of neurodegenerative diseases and cancer. There appears more evidence that cancer and neurodegenerative diseases may have common therapeutic targets [11]. One of them is NADH coenzyme [12]. Cancer cell metabolism requires increased glucose uptake and predominance of aerobic glycolysis for adenosine triphosphate production even in the presence of the sufficient amount of oxygen [13, 14]. Such reprogramming of energy metabolism may be used as a distinguishing feature of cancer [15]. The study [16] shows that increase in oxidative phosphorylation inhibits cancer growth *in vitro* and *in vivo*.

Increased glycolytic activity promotes enhanced production of lactate, which plays an important signaling role in tumor metabolism and may be involved in the inhibition of the anti-tumor immunity [17, 18]. In addition, the predominant use of glycolysis results in the increased ratio of the reduced to oxidized forms of NADH/NAD^+^ coenzyme in cancer cells relative to normal cells [19, 20]. Taking into account the ability of MB to interact with NADH, the use of MB for tumor therapy looks promising. Moreover, there are data in the literature about the interaction of MB with lactate. In the pioneering works devoted to the MB study as an antidote, it was supposed that its ability to increase oxygen uptake by the tissues is proportional to their rate of lactate production [21]. However, a detailed exploration of MB interaction with coenzymes was not carried out. In the present work, we investigated MB interaction with NADH, FADH_2_, and lactate, which were selected based on the key features of tumor metabolism.

Effective use of MB for anti-tumor therapy has been demonstrated on the cell cultures *in vitro*. It has been shown on blood cells that MB decreases the generation of lactic acid, which indirectly indicates switching of metabolism from glycolysis to oxidative phosphorylation [22]. The study on the glioblastoma cells has demonstrated that MB increases the rate of oxygen uptake, decreases lactic acid production and extracellular acidification rate, and inhibits proliferation [23].

Despite the promising results obtained *in vitro*, the *in vivo* studies are needed, since a tumor represents a complex heterogeneous structure including not only cancer but also immunocompetent cells and extracellular matrix. Our investigations have demonstrated the effect of MB on tumor oxygenation on the mouse model of Lewis lung carcinoma using intravenous introduction: after a short-time decrease, oxygenation level grew higher than initial value [24, 25]. Fluorescence lifetime imaging microscopy (FLIM) showed that already 5 min after MB injection, a shift on the NADH metabolic trajectory towards oxidative phosphorylation was noted.

In the present study, a long-term effect of MB on the tumor metabolism *in vivo* was investigated on the mouse model of Ehrlich carcinoma using its intravenous and oral administration. FLIM was employed to analyze the fluorescence lifetime of the free and bound forms of NADH coenzyme involved in glycolysis and oxidative phosphorylation. The possibility of tumor growth inhibition by the metabolic status correction has also been studied.

## Materials and Methods

### Reagents

1% methylene blue aqueous solution (active substance — methylthioninium chloride; Samarmedprom, Russia) was used for investigations. NADH disodium salt (100%, grade 1; Merk, Germany) was used to prepare NADH solution; FAD disodium salt hydrate (Sigma-Aldrich, USA) was used to prepare FADH_2_ solution; sodium lactate (Sigma-Aldrich, USA) was used to prepare lactate solution. Measurements were conducted in distilled water (PanEco, Russia).

### Absorption spectroscopy

Hitachi U3400 spectrophotometer (Hitachi, Japan) was employed to register absorption spectra in the range of 200–1000 nm in 2 mm pathlength quartz cuvettes. The absorption spectrum analysis allowed us to investigate the transition of the MB basic form to its reduced leucoform in interaction with other substances. Lactate was used at 10 mM concentration (a physiological concentration in pathological conditions). The concentration of NADH and FADH_2_ in the tested solutions was 0.25 mg/ml; MB concentration — 0.01 mg/ml. Absorption spectra were recorded immediately after mixing coenzyme and lactate solutions with MB and after 20 min.

### Evaluation of the MB effect on tumor metabolism in vivo

Male BALB/c mice aged 8–10 weeks weighing 25–30 g were used in the experiment. Mice were kept in standard cages at 21°C with 12:12-hour day–night cycle and a free access to standard laboratory chow and water. Ehrlich carcinoma was used as a model tumor. Experiments were carried out on day 12 after intramuscular tumor inoculation to the right hind leg.

The experiments on animals were approved by the Ethics Committee of N.N. Blokhin National Medical Research Center of Oncology (Moscow, Russia). Housing of animals and performance of the experiments were in compliance with the European Convention for the Protection of Vertebrate Animals used for Experimental and Other Specific Purposes (Strasburg, 2006).

Mice were divided into three experimental groups depending on MB concentration and administration route. Group 1 and 2 included mice with intravenous administration (i.v.) of MB at the dose of 10 and 20 mg/kg, respectively, on day 1 and 7 of the experiment. Group 3 received MB orally with drinking water (per os) at the daily dose of 10 mg/kg for 2 weeks. A negative control, i.e. the tumor without MB administration, served as a group of comparison.

The hemoglobin oxygenation *in vivo* was measured by the method of optical absorption of hemoglobin [26] with a halogenic lamp as a light source. Diffuse reflectance spectra were recorded using the LESA-01- Biospec fiber-optic spectrometer (BIOSPEC, Russia). The oxygenation was calculated as a ratio of oxygenated hemoglobin to the total hemoglobin absorption obtained from the absorption spectrum. Oxygenation was measured at five points both in the tumorous and normal muscular tissue for each mouse.

The mice were sacrificed on day 14 of the experiment. Tumors together with subcutaneous tissue, skin and muscles were removed by en-bloc resection and frozen. Cryosections were prepared using a freezing Microm HM 560 Cryostat microtome (Thermo Scientific, USA). 5 μm sections were examined on the laser scanning confocal LSM-710-NLO microscope (Carl Zeiss, Germany).

Time-resolved images for NADH autofluorescence and MB fluorescence were recorded at two-photon excitation with 740 nm wavelength using the femtosecond Chameleon Ultra II laser (Coherent, USA) with 140 fs pulse duration and 80 MHz repetition rate. Optical band filters FB450-40 (Thorlabs, USA) and BP 640/30 (Carl Zeiss, Germany) served to isolate fluorescence signals from NADH and MB, respectively. Images were processed using SPCImage 8.0 software (Becker & Hickl GmbH, Germany). Metabolic changes were evaluated by means of a1/a2 metabolic index calculated as a ratio of exponent coefficients of short and long NADH lifetime.

### Statistical data processing

The Shapiro–Wilk test was used for statistical analysis of the obtained data to determine the normality of sample distribution. If the distribution was normal, the two-sample t-test was performed. Groups were compared using the Student’s t-test. Data were considered statistically significant at p<0.05.

## Results

### Investigation of MB interaction with NADH, FADH_2_, and lactate

**Figure 1.**
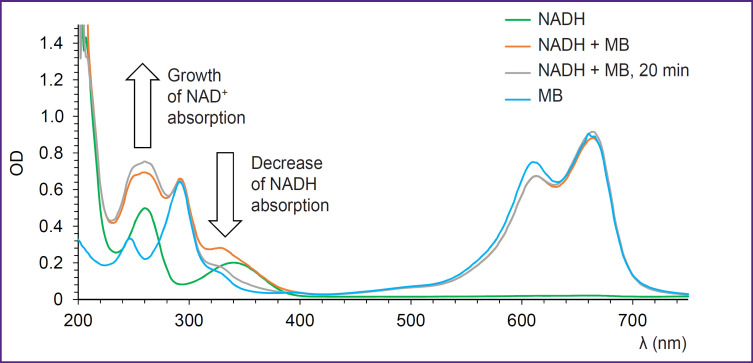
Absorption spectra recorded immediately and 20 min after the addition of MB to the NADH solution

Absorption spectra recorded after the addition of MB to the NADH solution are presented in Figure 1. As time passes, absorption in the region of 340 nm corresponding to NADH absorption decreases whereas absorption in 260 nm region corresponding to NAD^+^ grows. It means that reduction-oxidation reaction occurs between MB and NADH, in which NADH is an electron donor and MB is an acceptor. The reaction results in oxidation of NADH to NAD^+^. It is interesting to note that no reduction of MB absorption due to the transition to the colorless leucoform is observed. This effect is connected with aerobic conditions of the experiment. At the same time, rapid reoxidation of the leucoform by oxygen to MB takes place resulting in the constant amount of MB in the solution and absorption remains unchanged [27].

**Figure 2.**
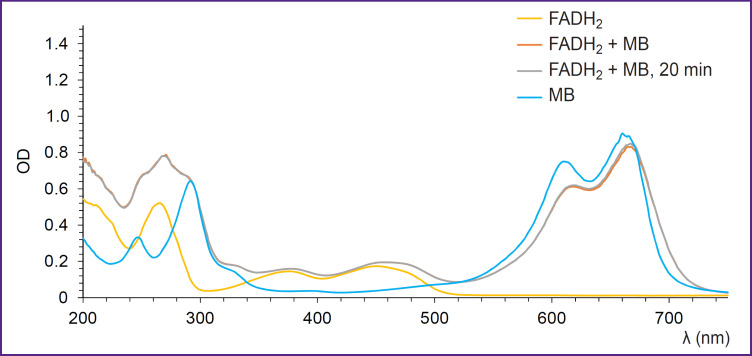
Absorption spectra recorded immediately and 20 min after the addition of MB to the FADH^2^ solution

Absorption spectra recorded after the addition of MB to the FADH_2_ solution are shown in Figure 2. It is seen on the obtained spectra that after the interaction of MB with FADH_2_, no changes in the spectrum shape in the region corresponding to FADH_2_ are observed. In addition, the spectrum in the region of FADH_2_ does not change with time. However, there is a shift of MB position maximum in the red part of the spectrum, which was not registered in the experiments *in vivo* [24, 25]. This indicates that the reaction with FADH_2_ coenzyme is not a key mechanism of MB action on tumor metabolism.

**Figure 3.**
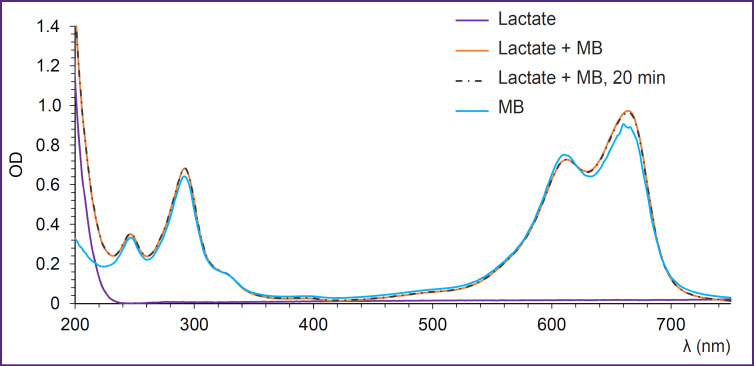
Absorption spectra recorded immediately and 20 min after the addition of MB to the lactate solution

A similar result has been obtained, when we studied the interaction of MB with lactate. Absorption spectra recorded after the addition of MB to the lactate solution are presented in Figure 3. Changes in the spectrum shape indicating the reaction process are not observed. The spectrum does not also change with time.

Thus, the results of spectroscopic investigation of MB with coenzymes and lactate made us come to the conclusion that NADH is the main coenzyme, with which interaction of MB takes place.

### The effect of MB on tumor metabolism in vivo: assessment of oxygenation and time-resolved fluorescence microscopy

**Figure 4.**
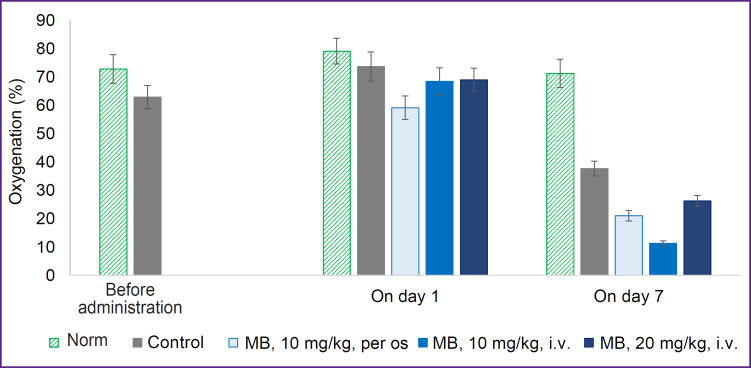
The degree of tumor oxygenation determined by hemoglobin absorption before therapy, on day 1 and day 7 after therapy with MB The value differences are statistically significant (p<0.05, Student’s t-test) between the norm and control before administration and between all groups on day 7

Before therapy, differences in oxygenation between the normal tissue and tumor (70 and 60%, respectively) were not large, since all the tumors were small in size, but statistically significant (Figure 4). No differences in oxygenation between the groups were actually observed on day 1 of the therapy. Oxygenation decreased in the group receiving MB with water, which was likely caused by the beginning of MB accumulation in the tumor after oral administration. On day 7, tumor oxygenation in the control group decreased considerably relative to the norm due to the tumor progression. In the groups with MB, oxygenation was lower than in the control group.

The most rapid tumor growth was observed in the control group; already on day 10, the tumor volume exceeded 1.5 cm^3^. In the group receiving MB with drinking water, the decrease of the tumor growth rate was noted in comparison with the control group, reaching the volume of 1.5 cm^3^ by day 14. In the groups with intravenous introduction, essential reduction of the tumor growth rate relative to the control has not been established. We suppose that in case of two-time injection of MB (on days 1 and 7), its action does not last too long to obtain a good therapeutic effect on the tumor. It should also be noted that when a high concentration of MB (20 mg/kg) was introduced intravenously, a pronounced vascular effect was also observed; some mice in the group were noted to have caudal vein thrombosis.

We assume that the result obtained may be explained by the MB influence on tumor metabolism. According to the results of our experiments [24, 25], 5 min after intravenous injection of MB, an increase in oxygen consumption by the tumor and a decrease in the oxygenation are observed. Along with this, a shift of metabolism towards oxidative phosphorylation was registered. One hour after MB introduction, oxygenation grew higher the initial values.

Thus, in this experiment, as long as MB is affecting the tumor, the measured oxygenation is lower, however, the tumor growth may be slower due to the metabolic shift relative to the control group without the action of MB.

**Figure 5.**
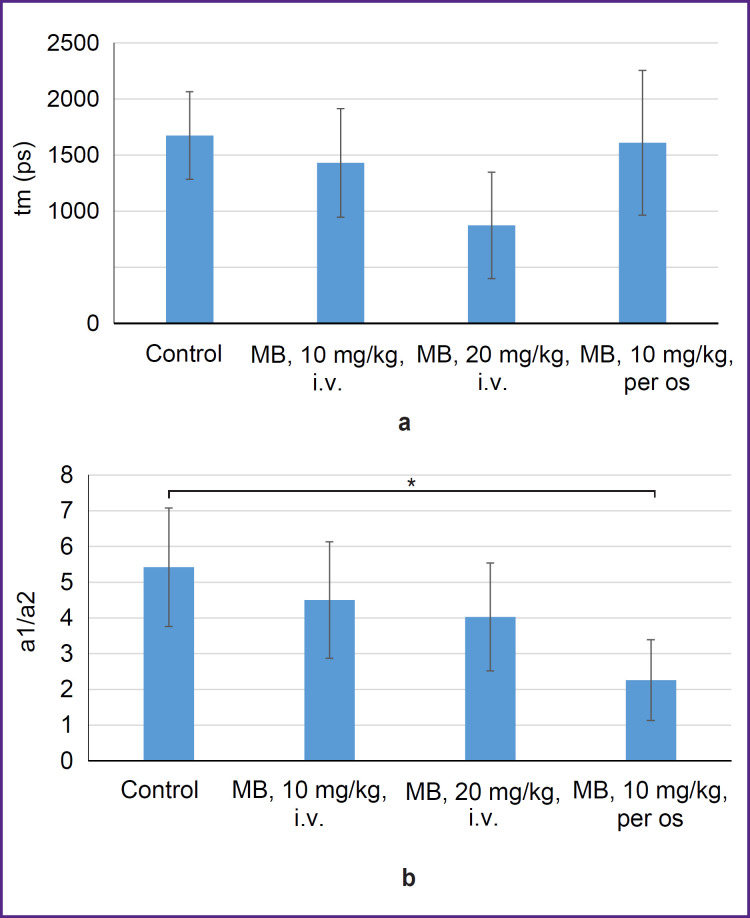
Mean lifetime tm (а) and a1/a2 metabolic index (b) for the groups with intravenous MB administration on day 1 and day 7 and the group receiving MB orally with drinking water for 2 weeks * Value differences are statistically significant (p<0.05, Student’s t-test)

This hypothesis was tested using FLIM microscopy. To assess the changes in metabolism, we used an average value of lifetime tm and a1/a2 metabolic index calculated as a ratio of exponent coefficients of the short and long NADH lifetimes (Figure 5).

Statistically significant differences for the average lifetime tm between the groups have not been established. The a1/a2 metabolic index was noted to decrease in the groups receiving MB therapy; statistically significant decrease relative to the control was observed for the group receiving MB with drinking water for 2 weeks. The decrease of the metabolic index is evidence of a shift from glycolysis to oxidative phosphorylation. This result confirms the previous assumption on a prolonged effect of MB on a tumor in case of the oral administration, which is expressed in the changes in the oxygenation level, metabolic shift towards oxidative phosphorylation, and reduction of the tumor progression rate. Although the obtained results are rather promising, the time of therapy and MB concentration need optimization in order to achieve a pronounced therapeutic effect.

## Discussion

NADH coenzyme is a vitally important molecule in all organisms and a key component in receiving energy by a cell from nutritional substances, and also in signaling pathways. NADH-dependent signaling pathways are multiple and diverse; they regulate transcription, DNA reparation, apoptosis, and metabolism. Many of these processes are connected with tumor progression, therefore correction of NADH metabolism is thought to be a promising therapeutic concept for cancer treatment [12, 19, 20].

One of the interesting approaches is the development and study of nanozymes, nanostructures, which imitate catalytic properties of natural enzymes. The majority of nanozymes can catalyze degradation of peroxides and are actively used to generate oxygen *in situ* [28]. Interest is growing in the development of nanozymes for other coenzymes involved in cellular metabolic redox reactions such as NADH/NAD^+^ [29]. The known redox staining agents, MB for example, may be used for the same purposes. In addition, a small molecular mass allows MB to be rapidly delivered to the tissues; when introduced intravenously, a high MB concentration was observed in the brain. It means that MB can pass through hematoencephalic barrier [30]. Such a high penetration capacity makes MB perspective from the point of view of accumulation in the tumors with impaired vascular network as well as in the necrotic center of rapidly growing solid tumors often with a strongly limited access of nutritional substances.

A great advantage is that MB is well-studied and has already been used in clinical practice and, therefore, does not require large toxicological investigations as in case with nanoparticles. Presently, MB is approved by the Food and Drug Administration (FDA, USA) for intravenous and oral administration for methemoglobinemia therapy and as a surgical dye. In 2025, there were registered 281 interventional studies (clinicaltrials.gov) worldwide to explore clinical MB utility in areas ranging from oncology to depression: of them 74 were in oncology, 141 clinical trials have been completed. A low cost of the agent and its effectiveness are also worth mentioning.

The results obtained by us have demonstrated an effective interaction of MB with NADH coenzyme. Interestingly that no direct interaction with lactate was observed. The decrease of the lactate amount and extracellular acidification rate after MB introduction, which is reported in the literature [31], is supposed to be a secondary effect mediated by the metabolic shift towards oxidation phosphorylation as a result of interaction of MB with NADH.

Another important result of our study is the demonstrated possibility of using MB for the correction of tumor metabolism and the decrease of its growth rate. Despite the promising results obtained, the time of therapy and MB concentration should be optimized to achieve pronounced therapeutic effect.

## Conclusion

The interaction of MB with NADH, FADH_2_ coenzymes, and lactate has been studied. NADH was established to be the main coenzyme, with which MB interacts.

The effect of MB on tumor metabolism *in vivo* and the possibility of inhibiting the tumor growth have been investigated. There was no essential reduction of the tumor growth rate in the groups with intravenous administration relative to the control. The decrease of the tumor growth rate compared to the control group as well as the reduction of the oxygenation level were observed in the group receiving MB with drinking water. The result obtained was explained by the MB impact on the tumor metabolism. The a1/a2 metabolic index statistically significantly decreased in the group receiving MB with drinking water for 2 weeks relative to the control group, indicating a shift from glycolysis to oxidative phosphorylation. Hence, while MB is acting on the tumor, the measured oxygenation is lower, however, due to the metabolic shift, the tumor growth in this case is slower relative to the control group, which is free from the MB influence.
